# HSV-2 Regulates Monocyte Inflammatory Response via the Fas/FasL Pathway

**DOI:** 10.1371/journal.pone.0070308

**Published:** 2013-07-29

**Authors:** Malgorzata Krzyzowska, Piotr Baska, Piotr Orlowski, Robert Zdanowski, Anna Winnicka, Kristina Eriksson, Wanda Stankiewicz

**Affiliations:** 1 Department of Preclinical Sciences, Faculty of Veterinary Medicine, Warsaw University of Life Sciences, Warsaw, Poland; 2 Military Institute of Hygiene and Epidemiology, Warsaw, Poland; 3 Department of Pathology and Veterinary Diagnostics, Faculty of Veterinary Medicine, Warsaw University of Life Sciences, Warsaw, Poland; 4 Department of Rheumatology and Inflammation Research, University of Gothenburg, Gothenburg, Sweden; INSERM-Université Paris-Sud, France

## Abstract

Monocytic cells represent important cellular elements of the innate and adaptive immune responses in viral infections. We assessed the role of Fas/FasL in promoting monocyte apoptosis during HSV-2 infection by using an in vitro model based on the murine RAW 264.7 monocytic cell line and an in vivo murine model of HSV-2 infection applied to C57BL6, MRL-*Fas^lpr^/J* (*Fas−/−*) and C3-*Fasl^gld^/J* (*FasL−/−)* mice. HSV-2 infection of the monocytic cell line led to early induction of apoptosis, with no protective expression of anti-apoptotic Bcl-2. HSV-2 infected monocytes up-regulated Fas and FasL expression early during in vitro infection but were susceptible to Fas induced apoptosis. The vaginal monocytes in the HSV-2 murine model of infection up-regulated FasL expression and were susceptible to Fas induced apoptosis. HSV-2 infection of Fas and FasL- deficient mice led to decreased apoptosis of monocytes and impaired recruitment of NK, CD4+ and CD8+ T cells within the infection sites. The vaginal lavages of HSV-2 infected Fas and FasL- deficient showed decreased production of CXCL9, CXCL10 and TNF-α in comparison to HSV-2 infected wild-type mice strain. The decreased recruitment of immune competent cells was accompanied by delayed virus clearance from the infected tissue. Triggering of the Fas receptor on HSV-2 infected monocytes in vitro up-regulated the expression of CXCL9 chemokines and the cytokine TNF-α. Our study provides novel insights on the role of Fas/FasL pathway not only in apoptosis of monocytes but also in regulating local immune response by monocytes during HSV-2 infection.

## Introduction

Herpes simplex virus 2 (HSV-2) is a sexually transmitted pathogen that infects the genital tract mucosa and causes the most common genital ulcer disease in humans. As for many other viruses, HSV-2 interferes with apoptotic programs in host cells. Clinical and laboratory adapted HSV-2 strains can block apoptosis in cells of epithelial and keratinocyte origin [1]–[3], showing the existence of an “apoptosis prevention window” [1] during the first 6 hours of HSV-2 infection. This suppression of apoptosis has been shown to result from the expression of the anti-apoptotic proteins Bcl-2, NF-κB and Akt [1]–[3]. On the other hand, HSV-2 mediated apoptosis has been documented to occur both in vivo in the neuronal tissue [4]- and in vitro – in lymphoid cells including monocytes [5], dendritic cells [6], the T-cell leukemia line Jurkat, the B cell lymphoma line Ramos and primary blood CD4+ T lymphocytes [7]. Induction of apoptosis by HSV-2 in the U937 monocytoid cells [5] was associated with down-regulation of the anti-apoptotic Bcl-2 protein, while its over-expression enabled sustained productive virus infection [8]. Apoptosis of murine peritoneal macrophages infected with HSV-2 was also observed [9].

Professional phagocytes (neutrophil granulocytes and monocytes/macrophages) constitute an important first line of defence against microbial intruders, including many viruses. Infiltration of monocytes/macrophages in the vaginal tissue can be detected during the acute stage of HSV-2 infection [10]. Monocytes are recruited to the infected genital mucosa and monocyte-derived antigen presenting cells are required to elicit interferon-γ (IFN-γ) secretion from effector Th1 cells in order to mediate antiviral protection during primary HSV-2 infection [11].

The Fas (Apo-1, CD95) cell-surface death receptor belongs to the tumor necrosis factor (TNF) receptor superfamily and mediates apoptosis upon binding of its natural ligand, FasL (CD178); the involvement of this receptor/ligand pair in induction of apoptosis is generally recognized [12]. However, evidence accumulates on Fas as a mediator of apoptosis-independent processes including proliferation, angiogenesis, fibrosis and inflammation [13], [14].

We have previously shown that HSV-2 infected keratinocytes and epithelial cells upregulated Fas and FasL, but were resistant to Fas induced apoptosis [2]. Furthermore, simultaneous stimulation of Fas receptor and HSV-2 infection blocked the production of pro-inflammatory chemokines and cytokines by infected cells. Therefore, it is possible that, in the context of HSV-2 infection, the Fas/FasL interaction may not only be limited to the elimination of HSV-2 infected cells via apoptosis, but also be related to the development of inflammatory lesions preceding the induction of local immune responses to the infection.

In this study, we investigated the role of Fas receptor in HSV-2 infected monocytes using a monocyte cell line and a well-established murine model of genital herpes in MRL-Fas(lpr)/J (*Fas−/−*), C3-Fasl(gld)/J (*FasL−/−*) and C57BL6 mice. Monocytes during in vitro and in vivo HSV-2 infection up-regulated FasL and were susceptible to Fas-induced apoptosis. Monocytes of Fas and FasL-deficient mice underwent delayed apoptosis and produced significantly less CXCL9, CXCL10 and TNF-α than monocytes in the wild type mice during HSV-2 infection. Furthermore, Fas and FasL-deficient mice showed impaired mobilization of NK, CD4+ and CD8+ T cells, and delayed virus clearance from the vaginal tissue. Our results suggest that Fas/FasL pathway may play a role in controlling the production of pro-inflammatory chemokines and cytokines by monocytes following HSV-2 infection, thus fine-tuning the genital immune response to HSV-2 infection.

## Materials and Methods

### Ethics Statement

This study was performed in strict accordance with the recommendations in the Polish Act of 21 January 2005 on animal experiments (OJ no. 33, item 289) and Directive 2010/63/EU of the European Parliament and the Council of 22 September 2010 on the protection of animals used for scientific purposes. The protocol was approved by the 3rd Local Committee on the Ethics of Animal Experiments in Warsaw, Poland (Permit Number: 58/2011).

### Virus

The HSV-2 strain 333 was grown and titrated in African green monkey kidney cells (GMK-AH1) and prepared by one cycle of freeze-thawing and subsequent removal of cellular debris by centrifugation [2].

### Cell Lines and in vitro HSV-2 Infection

The mouse monocyte RAW 264.7 cell line was purchased from ATCC (TIB-71) and mouse keratinocyte cell line 291.03C was kindly provided by M.Kulesz-Martin (Department of Dermatology, Oregon Health and Science University, Portland, USA). Malgorzata Krzyzowska obtained agreement for the transfer of 291.03C cell line for academic or research institution (MTA) from Oregon Health and Science University. 291.03C cell line is a published cell line [15]. RAW 264.7 cells were maintained in RPMI-1640 medium with 10% fetal bovine serum (FBS) and 1% antibiotics (Gibco by Life Sciences Technologies, Carlsbad, CA, USA). The 291.03C line, which is a 7,12-dimethylbenz[a]anthracene – initiated clone derived from non-transformed 291 cells [15], was cultured in D-MEM (Gibco) supplemented with 5% FBS (Gibco), 10 ng/mL epidermal growth factor (Sigma, St. Luis, MO, USA) and 1% antibiotic (Gibco). The cell lines were infected with HSV-2 333 strain at MOI = 1, incubated for up to 48 hours and then harvested by trypsinisation or scraping.

### Apoptosis Detection

Apoptosis was detected using Annexin V-Apoptosis detection kit I (BD Biosciences, Franklin Lakes, NJ, USA), according to the manufacturer’s protocol. The annexin V-positive, propidium iodide negative cells, were scored as apoptotic cells, while all propidium iodide positive cells were considered to be necrotic. For double staining of apoptotic and HSV-2 infected cells, M30 CytoDEATH kit was used (Roche, Indianapolis, IN, USA), according to the manufacturer’s protocol. To detect active caspase-9 form, polyclonal goat anti-cleaved caspase-9 p10 (h331) antibody was used (Santa Cruz Biotechnology, Santa Cruz, CA, USA). Prior to staining, the cells were permeabilised with Cytofix/Cytoperm kit (BD Biosciences), and then stained according to the manufacturer’s protocol. All stainings were analysed at the FACS Calibur (BD Biosciences) with CellQuest software (Le Pont De Claix, France).

### Antibodies and Flow Cytometric Analysis

For detection of Fas and FasL, cells were washed in 1% FBS/PBS, then FITC-conjugated hamster anti-mouse Fas antibody and PE-conjugated hamster anti mouse FasL antibody were used (BD Biosciences). Intra-cellular antigens were detected using Cytofix/Cytoperm fixation/permabilisation kit (BD Biosciences) and the following antibodies: PE-conjugated monoclonal hamster anti-Bcl-2 antibody and polyclonal rabbit anti-Bax antibody (BD Biosciences). HSV-2 antigens were detected using rabbit FITC-conjugated rabbit polyclonal anti-HSV-1/2 antibody (Dako, Glostrup, Denmark). Following incubation with primary antibodies, appropriate anti-rabbit PE antibodies were used, where necessary (BD Biosciences). The stained cell suspensions were analyzed in the FACS Calibur (BD Biosciences) for the percentage of positively stained cells and/or mean fluorescence intensity (MFI).

### HSV-2 Infection in Mice

Female mice, 6- to 8-week old, were used for all experiments. B6. MRL-Faslpr/J (*Fas−/−*) and B6Smn.C3-Faslgld/J (*FasL−/−*) mice were purchased from the Jackson Laboratory (Bar Harbor, ME, USA) and a breeding colony was maintained at the Oncology Centre (Warsaw, Poland) animal facilities. C57BL/6 mice were purchased from the Mossakowski Medical Research Centre (Warsaw, Poland) and used as wild-type controls. Mice were injected s.c. with 2.0 mg/kg of medroxyprogesterone (Depo-Provera; Upjohn Puurs-Belgium) in 100 µl of PBS. Five days later, the mice were infected by intravaginal inoculation with 10^4^ PFU/mouse of HSV-2 strain 333 in 25 µl of PBS. The animals were kept in ventilated cages under specific pathogen-free conditions at the Faculty of Veterinary Medicine at Warsaw University of Life Sciences. Three and seven days following intra-vaginal HSV-2 infection, the animals were killed and the vaginal tissue was isolated.

### Immunofenotyping of Animal Tissues

Cell suspensions from the vaginal tissue were prepared as follows: vaginal tissues from 3–5 mice were pooled and cut into small pieces and treated with collagenase-dispase (1 µg/ml)(Roche) in MEM medium at 37C° for 40 min. Treated tissues were pressed through a 70 µm cell strainer and washed in PBS/1%FBS. Monocytes were detected using hamster anti-CD11c-APC (HL3), rat anti-Ly-6G-PE (1A8) and rat anti-CD11b-FITC or PerCP (M1/70) antibodies (BD Biosciences), Fas and FasL were detected as described above, using FITC-conjugated antibodies, T cells were detected using rat anti-CD3e-FITC (145-2C11), rat anti-CD4-PE (RM4-5), rat anti-CD8-PE (53–6.7.) antibodies and NK cells were detected with rat anti-NK1.1-APC (PK136) antibody. For all phenotyping, PE-conjugated rat IgG2a or FITC-conjugated rat IgG2b and APC conjugated hamster IgG1 or rat IgG2a isotype antibodies were used (BD Biosciences). The intracellular stainings for TNF-α, CXCL9 and CXCL10 in monocytes were detected using the Cytofix/Cytoperm fixation/permabilisation kit with BD GolgiPlug (Brefeldin A) (BD Biosciences) in the vaginal tissue cells suspension using the antibodies described above. TNF-α, IL-1β, CXCL9 and CXCL10 were detected with the following antibodies: polyclonal rabbit anti-MIG (N-16; CXCL9), polyclonal goat anti-IP-10 (C19; CXCL10) (Santa Cruz), PE-conjugated monoclonal rat anti-TNF-α antibody (TN3-19.12; BD Biosciences) and hamster anti-IL-β antibody (BD Biosciences). Following incubation with primary antibodies, appropriate anti-rabbit PE or FITC-conjugated anti-goat and anti-hamster antibodies were used, where necessary (BD Biosciences). The stained cell suspensions were analyzed in FACS Calibur for the percentage of positively stained cells. The total cell count was set at 100.000 events.

For immunofluorescence staining, vaginal tissue was removed, fixed in 2% paraformaldehyde in PBS for 4 h, then washed twice in 10% sucrose/PBS before freezing and cryo-sectioning. Slides were stained with rat biotin-conjugated anti-CD11b antibody and rabbit polyclonal anti-HSV-2 antibody (Dako) in 1% goat serum in PBS/0.1% Triton X-100 for 30 minutes. This procedure was followed by 30 minutes incubation with goat anti-rabbit IgG-PE antibody (Dako) and FITC conjugated streptavidin. The slides were mounted in medium containing Hoechst 33342 (1 µg/ml) and the fluorescence was captured with Leica flourescence microscope equipped in Hamatsu C4880 cold CCD camera. For all stainings, isotype control antibodies - rat IgG1 and rabbit polyclonal IgG were used (BD Biosciences).

### Measurement of Cytokines

Concentrations of cytokines from culture supernatants and in vaginal lavages were determined using Mouse IL-1β and Mouse IP-10 Platinum ELISA kits (e-Biosciences, San Diego, CA, USA), mouse CXCL1 ELISA kit (Sigma-Aldrich) and Mouse Inflammation Cytometric Bead Array (CBA) and MIG Flex Set reagents (BD Biosciences, San Diego, CA) according to the manufacturer’s protocol. The results are presented as the means of assays performed in triplicates. Data were analyzed by using FCAP Array 0.1 and BD Cytometric Bead Array 1.4 software assay.

### Co-culture of Keratinocytes and Monocytes

Mouse peritoneal monocytes were isolated from B6. MRL-Faslpr/J (*Fas−/−*) and B6Smn.C3-Faslgld/J (*FasL−/−*) and C57BL6 mice and plated in RPMI-1640 medium with 10% FBS at 37C°. After 4 h of incubation, the non-adherent cells were discarded, adherent cells washed with medium, then collected using a cell scraper and placed at a ratio of 1∶5 in the cultures of 291.03C cell line at 12 h of HSV-2 infection. After 12 h of co-culture, the cells were collected and stained with anti-CD11b-FITC antibody and annexin V-PE kit, as described above, and analysed in FACS Calibur.

### Virus Titration by RT-PCR

The virus titers were determined as described previously [2]. Briefly, a 118-nucleotide segment of the gB region from HSV-2 region was amplified using a HSV-2 probe labeled with JOE (6-carboxy-4′,5′-dichloro-2′,7′-dimethoxyfluorescein) in a real-time PCR instrument ABI Prism 7000 (Applied Biosystems, Carlsbad, CA, USA) and titrated.

### Statistical Methods

Quantitative data were presented as means ± S.E.M. In the case of normal distribution of values, statistical comparisons were performed using the Student’s t-test. For data following non-Gaussian distributions, non-parametric Wilcoxon test was applied. In every analysis values of P<0.05 were considered significant.

## Results

### HSV-2 Infection Induces Early Apoptosis in Monocytes

**Figure 1:**
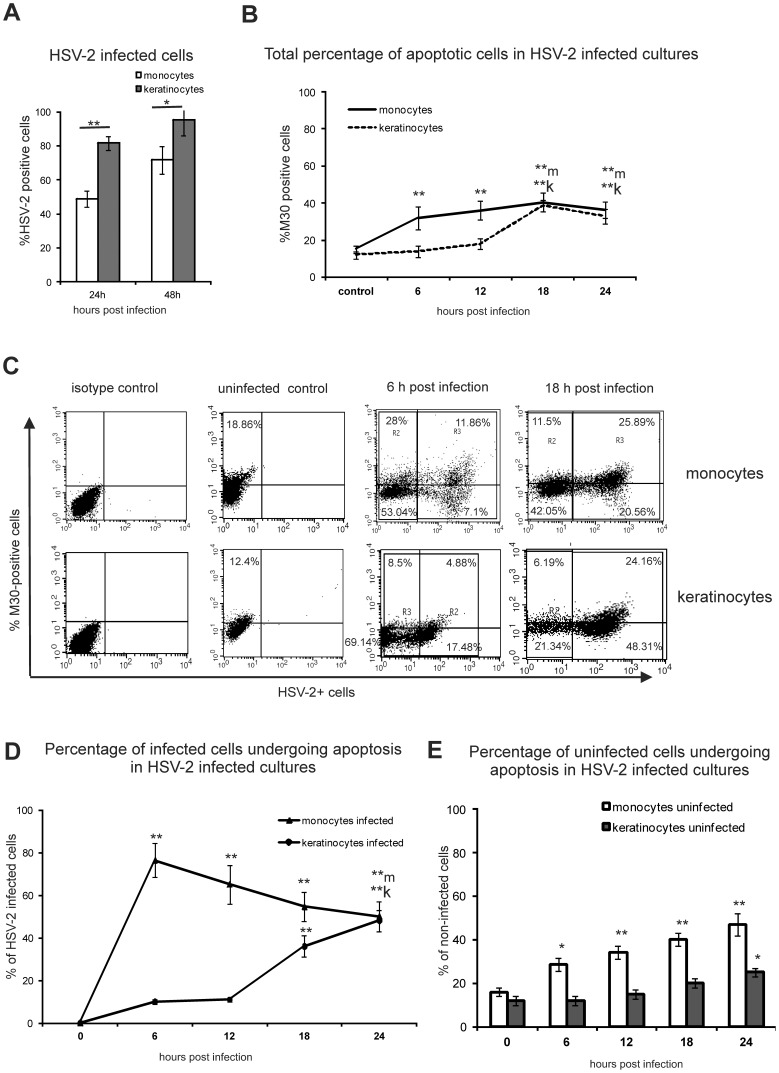
HSV-2 infection of mouse monocytes leads to early induction of apoptosis in comparison to keratinocytes. (A) HSV-2 infected monocyte cell culture showed significantly less infected cells at 24 and 48 h p.i. (hours post infection) in comparison to HSV-2 infected keratinocyte cell cultures. (B) Kinetics of total percentage of M30-positive (apoptotic) cells in monocyte RAW 264.7 and keratinocyte 03C cell line cultures during 24 hours after infection with HSV-2. (C) Representative dot plots showing M30 and/or HSV-2 positive cells detected by flow cytometry at 6 and 18 h p.i. in monocyte and keratinocyte cultures and in control uninfected cultures. (D) Kinetics of HSV-2 infected cells undergoing apoptosis in monocyte RAW 264.7 and keratinocyte 03C cell line cultures during 24 hours after infection with HSV-2 (R3 gate from panel C). (E) Kinetics of uninfected cells undergoing apoptosis in monocyte RAW 264.7 and keratinocyte 03C cell line cultures during 24 hours after infection with HSV-2 (R2 gate from panel C). Each bar represents the mean of 5 independent experiments (N = 5) ± SEM. * represents significant differences with *p*≤0.05, while ** means *p*≤0.001. *k means significant differences for keratinocyte cultures, while *m means significant differences for monocyte cultures.

Taking into account our previous results showing that HSV-2 infected mouse keratinocytes are resistant to apoptosis during the early phase HSV-2 infection, we decided to use the same keratinocyte cell line - 291.03C as a model of resistance to apoptosis during HSV-2 infection. Interestingly, both cultures of monocyte (RAW264.7) and keratinocyte (291.03C) cell lines infected with HSV-2 at the same multiplicity of infection (M.O.I.) showed different percentages of HSV-2 infected cells: 81.58±4.01 and 95.2±9.02% of keratinocytes at 24 and 48 h p.i. (hour post infection), respectively, and 48.76±4.75 and 71.69±8.98% of monocytes were HSV-2 infected at the same time points (Fig. 1A). At 18 and 24 h post infection (p.i.), both cultures showed similar percentages of cells with apoptosis-specific cytokeratin 18 cleavage (M30-positive cells) in comparison to uninfected control cultures (Fig. 1 B, C). However, during the first 12 hours of HSV-2 infection, significantly higher percentages of apoptotic cells were found in HSV-2-infected mouse monocytes cell line (RAW264.7) in comparison to HSV-2 infected keratinocyte (291.03C) cell line (*p*≤0.001) (Fig. 1B, C). HSV-2 infected monocyte cultures showed significantly more total percentage of apoptotic cells already at 6 and 12 h p.i. in comparison to uninfected control cells (*p*≤0.001) (Fig. 1B, C).

Within the population of infected cells, 50.5±7.1% of the HSV-2-infected monocytes and 48.23±4.98% of the HSV-2 infected keratinocytes showed apoptotic features at 24 h p.i. (Fig. 1D), while at 48 h p.i. the population of apoptotic and HSV-2 infected monocytes and keratinocytes accounted to 31±3.09 and 34±5.1% of cells (*p*≤0.01) (data not shown). When comparing HSV-2 infected monocytes and keratinocytes earlier during infection (6–18 h p.i.), significantly more HSV-2 infected apoptotic cells were observed in the monocyte cultures then in keratinocyte cultures (*p*≤0.001) (Fig. 1D). In fact, 76.5±7.99, 65.12±8.98 and 54.68±7.07% of HSV-2 infected monocytes at 6, 12 and 18 h p.i. were apoptotic, respectively in comparison to cultures of HSV-2 infected keratinocytes (*p*≤0.001) (Fig. 1D), where apoptosis levels increased no earlier than at 18 h p.i. (36±4.97% of HSV-2-positive cells, *p*≤0.05, Fig. 1D). The uninfected monocytes from the HSV-2 infected cultures were undergoing apoptosis already at 6 h p.i. (*p*≤0.05) (Fig. 1E), while uninfected keratinocytes were not undergoing significant levels of apoptosis until 24 h p.i. (*p*≤0.05) (Fig. 1E). These results indicate that both HSV-2 infected and uninfected monocytes are significantly more sensitive to apoptosis than uninfected cells or infected keratinocytes.

**Figure 2:**
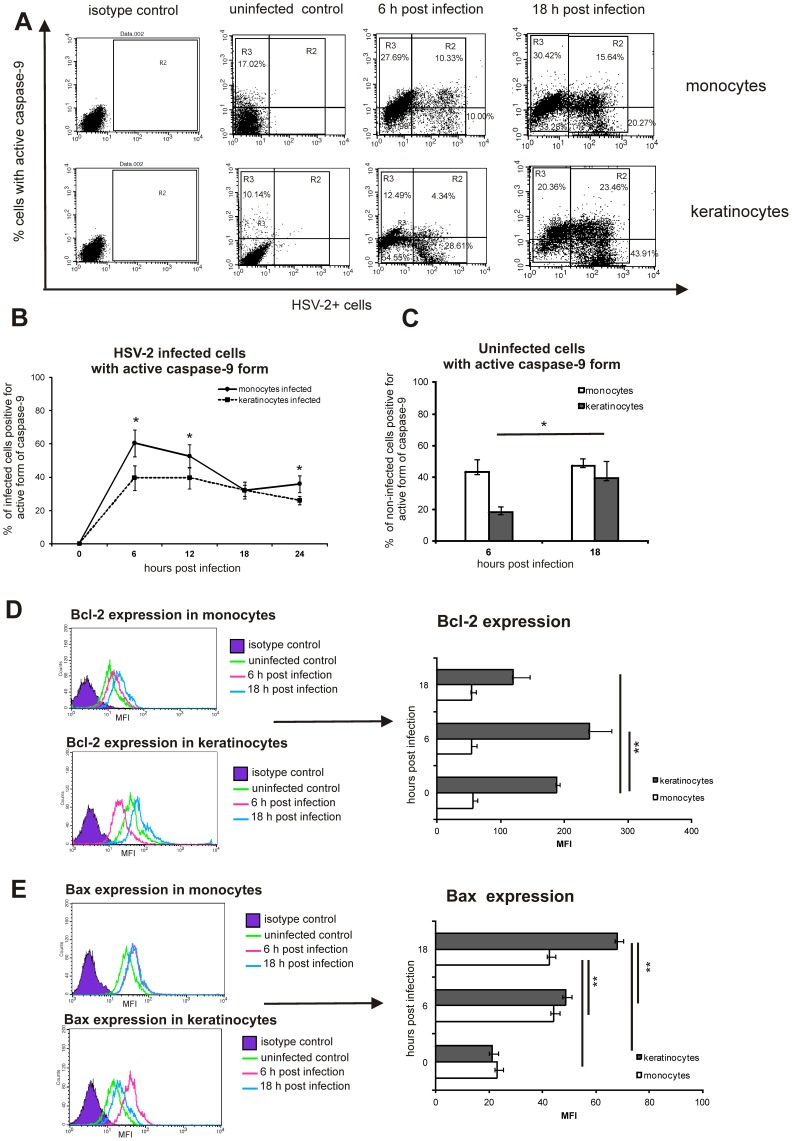
Monocytes do not up-regulate Bcl-2 expression during HSV-2 infection and show significant early caspase-9 activation. (A) Representative dot plots showing cells positive for HSV-2 and/or active form of caspase-9 detected by flow cytometry at 6 and 18 h p.i. and in control uninfected cultures of monocytes and keratinocytes. (B) Percentage of HSV-2-positive cells with active caspase-9 form in HSV-2 infected monocyte RAW 264.7 and keratinocyte 291.03C cell cultures (R2 gate from panel A). (C) Percentage of uninfected cells with active caspase-9 form in HSV-2 infected monocyte and keratinocyte cell cultures (R3 gate from panel A). (D), (E) Mean fluorescence intensity (MFI) showed as histograms (left panels) and bars (right panels) for Bcl-2 (D) and Bax (E) proteins expression in the monocyte (white bars) and keratinocyte (black bars) cell cultures infected with HSV-2 at 6 and 18 hp.i., detected by intracellular staining and flow cytometry. Controls are uninfected cultures (0 h p.i.). Each bar represents the mean from 5 experiments (N = 5) ± SEM. * represents significant differences with *p*≤0.05, while ** means *p*≤0.001.

To elucidate the role of mitochondrial pathway in apoptosis induction during HSV-2 infection, we looked for active form of caspase-9 in HSV-2 infected cell cultures. The total percentage of cells with active form of caspase-9 in HSV-2 infected keratinocyte cultures significantly increased at 18 h p.i. (58.33±4.83%) (*p*≤0.01) (Fig. 2A) in comparison to uninfected cultures, where the mean level of cells with active caspase-9 was 19.39±2.1% during all tested period. In contrast, HSV-2 infected monocyte cultures showed early activation of caspase-9 already at 6 h p.i. when approximately 50% of cells in cultures were positive for the active form of caspase-9 and this proportion remained significant until 18 h p.i. in comparison to uninfected control cultures (*p*≤0.001) (Fig. 2A). When analysing the populations of HSV-2 infected monocytes and keratinocytes for the activation of caspase-9, we found that significantly more HSV-2 infected monocytes showed activation of caspase-9 then HSV-2 infected keratinocytes at 6, 12 and 24 h p.i. (*p*≤0.05) (Fig. 2B), while at 18 h p.i. we observed the same percentage of HSV-2 infected cells with active form of caspase-9 in both tested cultures (Fig. 2B).

The uninfected monocytes in HSV-2 infected cultures showed a constant percentage of uninfected cells with active caspase-9 form (43.16±8.43% and 47.37±4.6% at 6 and 18 h p.i., respectively) (Fig. 2C). The percentage of uninfected keratinocytes with the active form of caspase-9 in HSV-2 infected cultures significantly increased from 17.95±4% at 6 h p.i. to 39.31±6.78% at 18 h p.i. (*p = *0.03) (Fig. 2C), when comparing both time points.

Since activation of caspase-9 is regulated by the family of pro- and antiapoptotic proteins from Bcl-2 family, here we assessed the HSV-2 infected monocytes and keratinocytes for the expression of anti-apoptotic Bcl-2 and pro-apoptotic Bax proteins; we found that HSV-2 infected cultures of monocytes did not up-regulated Bcl-2 expression in contrast to keratinocytes (Fig. 2D), thus rendering these cells susceptible to apoptosis via mitochondrial pathway. Both HSV-2 infected keratinocyte and monocyte cultures significantly up-regulated Bax expression already at 6 h p.i. (*p*≤0.001) (Fig. 2E) with keratinocytes showing the highest Bax expression at 18 h p.i. (*p*≤0.001) (Fig. 2E).

### HSV-2-infected Monocytic Cells Up-regulate FasL but Remain Sensitive to Fas Mediated Apoptosis

**Figure 3:**
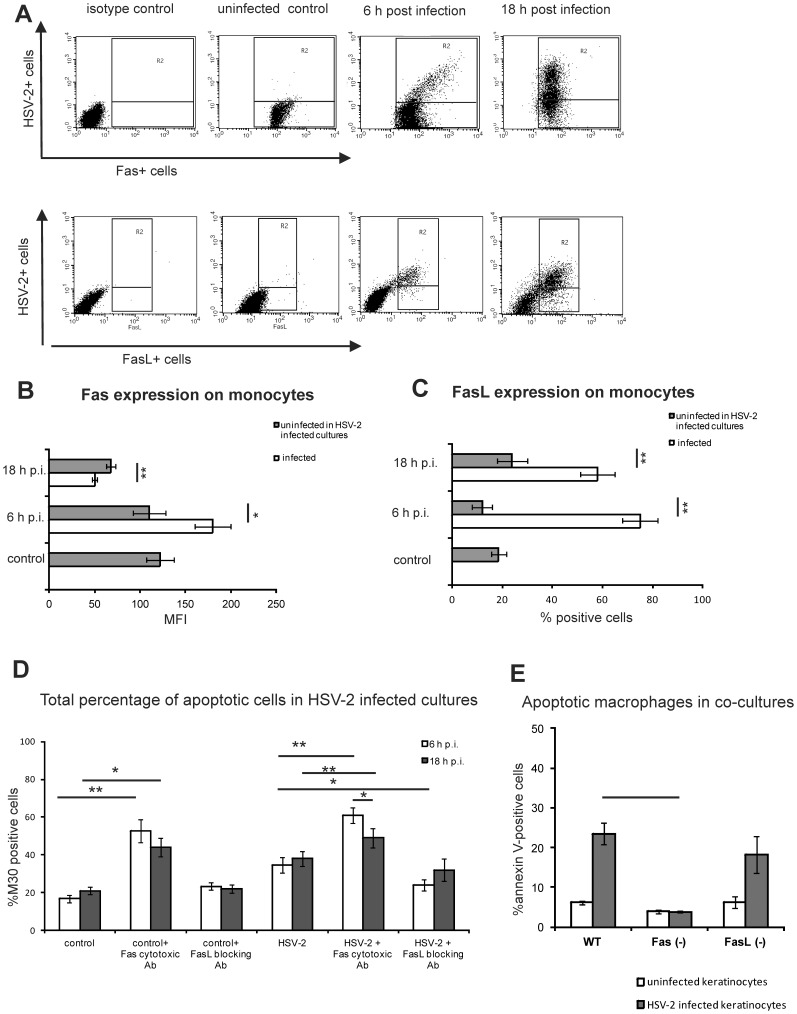
HSV-2 infected monocytes up-regulate FasL, but are susceptible to Fas-induced apoptosis. The cells in the HSV-2 infected RAW 264.7 cultures were analysed for Fas and FasL expression using flow cytometry and all event from gate R2 were divided into infected and uninfected cells expressing Fas or FasL. Fas expression is shown in the form of representative dot plots (A) or as the mean fluorescence intensity (MFI) for infected (white bars) and uninfected cells (grey bars) (B). FasL expression is presented as the representative dot plots (A) or as the percentage of FasL - positive cells (C). (D) Susceptibility to Fas-induced apoptosis was tested in RAW 264.6 cultures at 6 and 18 hours after infection with MOI = 1 in the presence of cytotoxic anti-Fas antibody (10 µg/ml) able to induce apoptosis through binding Fas receptor or FasL-blocking antibody (10 µg/ml). Apoptosis was detected using intracellular M30 staining. (E) Apoptotic monocytes (annexinV+/CD11b+ cells) after 12 h of co-culture with murine 291.O3C keratinocytes uninfected or HSV-2 infected at 18 h p.i. Each bar represents the mean from 3 experiments (N = 3) ± SEM. * represents significant differences with *p*≤0.05, while ** means *p*≤0.001.

Since we found that all control, uninfected RAW 264.7 monocytes express Fas, in order to visualize changes in Fas expression, we show Fas expression as mean fluorescence intensity (MFI). At 6 h p.i. HSV-2 infected monocytes significantly increased MFI for Fas in comparison to uninfected cells from cultures (*p = *0.02) (Fig. 3A, B). Interestingly, at 18 h p.i. a significant down-regulation of Fas expression was observed on both infected and uninfected monocytes present in HSV-2 infected cultures in comparison to 6 h p.i. (*p*≤0.001) (Fig. 3A, B). Infected cells showed significantly lower MFI for Fas in comparison to uninfected cells (*p*≤0.001) (Fig. 3B). Only approximately 20% of control cells are expressing FasL (Fig. 3A, C). During HSV-2 infection, FasL expression significantly increased only on HSV-2 infected monocytes at 6 and 18 h p.i. (*p*≤0.001) (Fig. 3C).

To assess the sensitivity of HSV-2-infected monocytes to Fas-induced apoptosis we used an anti-mouse Fas cytotoxic antibody (Jo-1 clone) and FasL-blocking antibody (MFL-4 clone). In the presence of Fas cytotoxic antibody at 6 and 18 h p.i. of HSV-2 infection, we detected significantly more apoptotic cells in comparison to infected cultures (*p*≤0.05) (Fig. 3D). Significantly more cells were undergoing apoptosis in the HSV-2 infected cultures in the presence of Fas cytotoxic antibody at 6 h p.i. in comparison to 18 h p.i. (*p = *0.05) (Fig. 3D). The presence of FasL-blocking antibody had influence on apoptosis induction in HSV-2 infected monocytes cultures only at 6 h p.i. (*p*≤0.05) (Fig. 3D).

Additionally, we applied a co-culture model to study apoptosis induced by FasL provided by HSV-2 infected 291.03C mouse keratinocytes in monocytes isolated from mice without the Fas gene (B6.MRL-Fas<lpr>/J) or without the FasL gene (B6Smn.C3-Fasl<gld>/J) and compared with the monocytes isolated from the background strain (C57BL/6). Monocytes isolated from the mouse strains were added to HSV-2 infected 291.03C mouse keratinocytes at 18 h p.i., when keratinocytes up-regulated both Fas and FasL, as shown previously [2]. The results showed that only monocytes isolated from Fas *(−/−)* strain showed significantly less apoptosis induction in comparison to monocytes isolated from FasL *(−/−)* and wild type strain (*p = *0.001) (Fig. 3E), albeit lack of Fas expression did not completely protected from apoptosis induction.

### Fas Influences Monocyte Involvement at the Inflammatory Lesions in vivo

**Figure 4:**
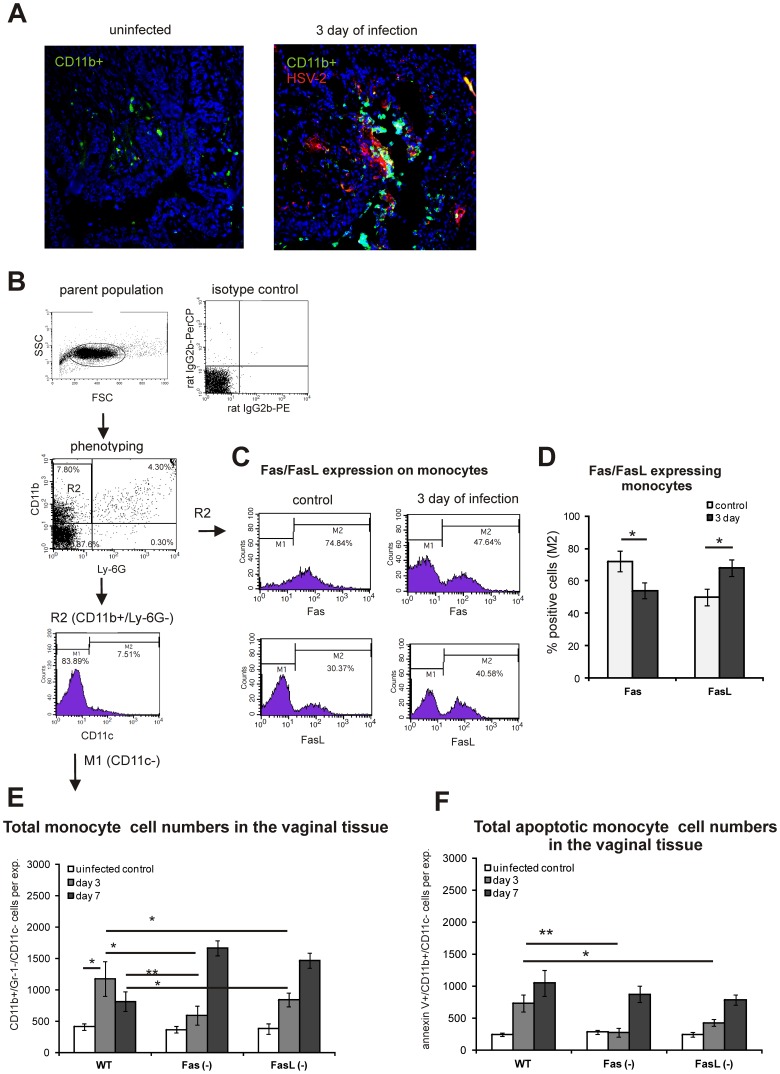
Monocytes during in vivo HSV-2 infection are susceptible to Fas-induced apoptosis. (A) CD11b+ cells (green) and HSV-2 antigen localisation (red) in the vaginal tissue isolated from control, uninfected mice C57BL/6 mice (left) and at 3 days of HSV-2 infection (right). The nuclei were counterstained with Hoechst 33342 (blue). (B) Gating strategy for identification of monocytes in the vaginal tissue (CD11b+/Ly6G−/CD11c-). Histograms (C) and bars (D) showing total percentage of Fas and FasL-expressing monocytes (CD11b+/Ly6G-) in the cell suspensions prepared from HSV-2 infected C57BL/6 mice at 3 day of infection and from control uninfected mice. (E) Total counts of monocytes (CD11b+/Ly6G−/CD11c-) in cell suspensions prepared from the vaginal tissues isolated from Fas *(−/−)*, FasL *(−/−)* and WT (C57BL/6) mice at 3 and 7 day of HSV-2- infection and from control uninfected mice. (F) Total counts of annexin V-positive monocytes in the vaginal tissue cell suspensions prepared from HSV-2-infected Fas *(−/−)*, FasL *(−/−)* and WT (C57BL/6) mice at 3 and 7 day of infection. The bars represent mean from 5 separate experiments ± SEM. * represents significant differences with *p*≤0.05, while ** means *p*≤0.01.

During HSV-2 infection of C57BL/6 mice, monocytes, both infected and uninfected, were detected within the infectious inflammatory lesions situated within epidermis of the vaginal tissue (Fig. 4A). Monocytes isolated from HSV-2 infected wild-type mice at 3 day of infection (CD11b+/Ly6G-) significantly down-regulated Fas and up-regulated FasL expression (*p*≤0.01) (Fig. 4B, C, D). During HSV-2 infection of C57BL/6 mice, the numbers of monocytes defined as CD11b+/Ly6G−/CD11c- (Fig. 4B) were significantly increased at 3 and 7 day of infection (*p*≤0.05) (Fig. 4E), albeit the total counts of monocytes at 7 day of infection were lower than at day 3 (Fig. 4E). To estimate how the presence of Fas and FasL proteins influences the monocyte frequency during HSV-2 infection, we infected Fas *(−/−)* and FasL *(−/−)* mice together with the background strain (C57BL/6). The control, uninfected mice of all tested strains showed no differences in the total numbers of monocytes (Fig. 4E). In both HSV-2 infected Fas *(−/−)* and FasL *(−/−)* mice, the total numbers of all monocytes at 3 day of infection increased similarly to wild-type C57BL6 mice (*p*≤0.01) (Fig. 4E). However, at 3 day of infection, the total numbers of all monocytes in Fas *(−/−)* and FasL *(−/−)* mice were significantly decreased in comparison to infected wild-type mice (*p*≤0.01) (Fig. 4E). On the contrary, at 7 day of infection, Fas and FasL-deficient mice showed increased numbers of monocytes in comparison to HSV-2 infected wild-type mice (*p*≤0.01) (Fig. 4E). To assess the involvement of Fas and FasL on apoptosis of monocytes during HSV-2 infection, we quantified apoptotic monocytes (annexin V+/CD11b+/Ly-6G-) (Fig. 4F). In all mice strains, the total counts of apoptotic monocytes increased significantly following infection; however, at 3 day of infection, the total numbers of apoptotic monocytes in the infected Fas *(−/−)* and FasL *(−/−)* mice were significantly lower (*p*≤0.01) (Fig. 4F). This tendency was observed also at 7 day of infection, although without significant differences between the tested strains (Fig. 4F).

### Fas is Involved in the Regulation of Monocyte Inflammatory Response in vitro and in vivo

**Figure 5:**
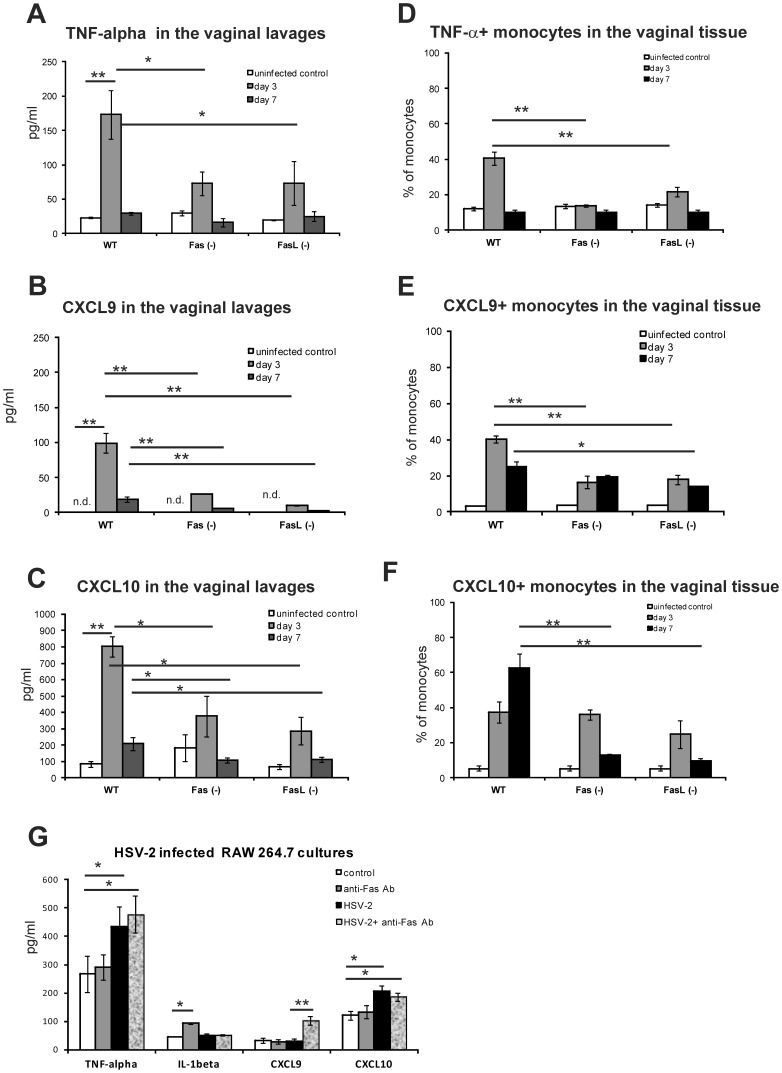
Fas is involved in production of TNF-α, CXCL9 and CXCL10 during HSV-2 infection. Vaginal tissues and vaginal lavages of Fas *(−/−)*, FasL *(−/−)* and WT (C57BL/6) mice (n = 5 mice per strain) infected with HSV-2 were collected at 3 and 7 day of infection. Uninfected mice were used as controls. The pooled vaginal tissues were used to prepare single cell suspensions and further stained for monocytes and intracellular expression of TNF-α, CXCL9 and CXCL10. Vaginal lavages from single mouse were used for further determination of TNF-α (A), CXCL9 (B) and CXCL10 (C). Percentages of monocytes with TNF-α (D), CXCL9 (E) and CXCL10 (F) expression in the vaginal tissues. (G) TNF-α, IL-1β, CXCL9 and CXCL10 levels in culture supernatants at 18 h p.i. of RAW 264.7 cell cultures infected with HSV-2 and exposed or not, to anti-Fas cytotoxic antibody (10 µg/ml). The bars represent the mean from 3 separate experiments (N = 3) ± SEM. * represents significant differences with *p*≤0.05, **p≤0.01. n.d. – not detected.

To further check how Fas and FasL influence the cytokine and chemokine production in vivo we assessed vaginal lavages for the production of chemokines (CXCL1, CXCL9 and CXCL10) and interleukins (IL-1β and TNF-α) at 3 and 7 day of infection. We observed that all tested mouse strains [C57BL6, Fas (−) and FasL (−)] at 3 day of HSV-2 infection significantly up-regulated production of CXCL1, CXCL9, CXCL10, IL-1β and TNF-α (*p*≤0.01) in the vaginal lavages in comparison to control, uninfected mice. However, upon infection, Fas- and FasL-deficient mice showed significantly less TNF-α production in the vaginal lavages at 3 day of infection in comparison to wild-type C57BL6 mice (*p*≤0.05) (Fig. 5A). The Fas and FasL- deficient mice showed significantly less CXCL9 production in the vaginal lavages at 3 and 7 day of infection in comparison to HSV-2 infected wild-type strain (*p*≤0.01) (Fig. 5B). Furthermore, Fas and FasL-deficient mice showed significantly lower levels of CXCL10 production at 7 day of infection in comparison to HSV-2 infected wild-type strain (*p*≤0.01) (Fig. 5C). We found no differences for CXCL1 and IL-1β levels in the vaginal lavages during HSV-2 infection in all tested strains (data not shown). Furthermore, we tested the vaginal tissue for the presence of monocytes expressing TNF-α, CXCL9 and CXCL10 (Fig. 5 D, E, F). We observed a significantly decreased percentage of TNF-α- expressing monocytes in HSV-2 infected Fas and FasL-deficient mice at 3 day (*p*≤0.01), but not at 7 day of infection (Fig. 5D) in comparison to HSV-2 infected wild-type strain. Similarly, we found significantly less percentages of CXCL9 - expressing monocytes in HSV-2 infected Fas and FasL-deficient mice at 3 day of infection (*p*≤0.01), but only in FasL-deficient mice at 7 day of infection (*p*≤0.05) (Fig. 5E). Fas and FasL-deficient mice showed significantly less percentages of CXCL10 - expressing monocytes at 7 day of infection (*p*≤0.01) (Fig. 5F) in comparison to HSV-2 infected wild-type strain.

To further assess whether stimulation through the Fas receptor influences the induction of inflammatory responses in HSV-2 monocyte cultures in vitro, we evaluated IL-1β, TNF-α, CXCL9 and CXCL10 levels in cell culture supernatants from HSV-2 infected cultures in the presence of Fas cytotoxic antibody triggering apoptosis through Fas. In response to HSV-2 infection, monocytes significantly up-regulated production of TNF-α and CXCL10 at 18 h p.i. (*p*≤0.05) (Fig. 5G). Stimulation of uninfected monocytes with anti-Fas antibody influenced only IL-1β production (*p = *0.01) (Fig. 5G). Addition of cytotoxic Fas antibody to HSV-2 infected cultures significantly induced expression of CXCL9 in comparison to HSV-2 infected cultures (*p = *0.01) (Fig. 5G).

### Lack of Fas or FasL Impairs Antiviral Immune Responses in the Vaginal Tissue

**Figure 6:**
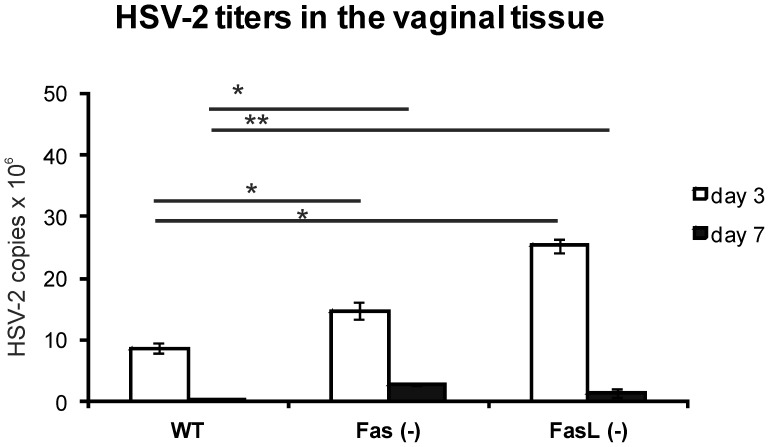
Lack of Fas and FasL expression results in delayed virus clearance from the infected tissue. HSV-2 DNA titers (copies/µg DNA) in the whole vaginal tissues of Fas *(−/−)*, FasL *(−/−)* and WT (C57BL/6) mice (n = 5 per strain) at 3 and 7 day of HSV-2 infection. The bars represent the mean from 3 separate experiments (N = 3) ± SEM. * represents significant differences with *p*≤0.005, **p≤0.001.

**Figure 7:**
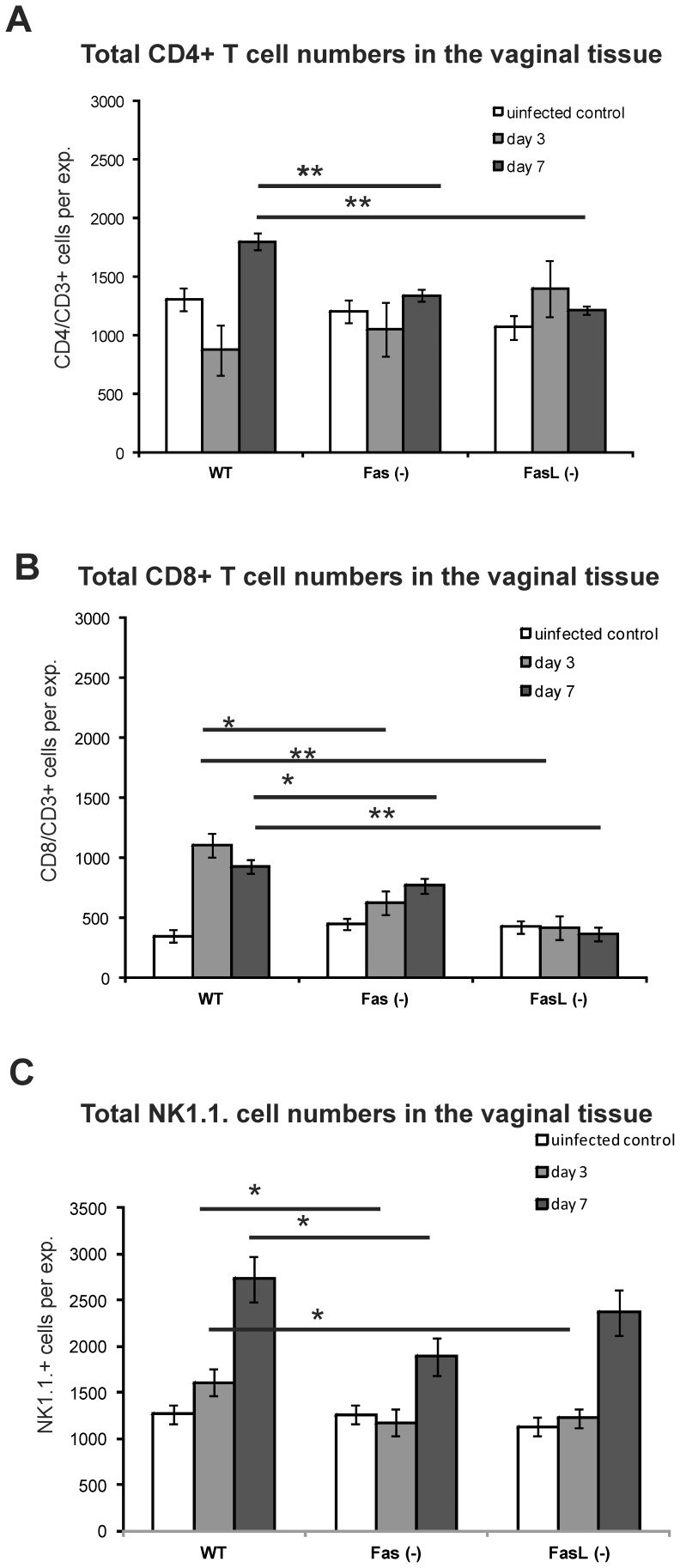
Decreased recruitment of NK, CD4+ and CD8+ T cells in HSV-2 infected Fas and FasL-deficient mice. Vaginal tissue samples from Fas *(−/−)*, FasL *(−/−)* and WT (C57BL/6) mice (n = 5 mice per strain) isolated at 3 and 7 day of HSV-2 infection were pooled and used to prepare single cell suspensions and further analyzed for the total numbers of CD4+ T cells (A), CD8+ T cells (B) and NK cells (C). Uninfected mice were used as controls. Each bar represents the mean total cell numbers from 5 separate experiments (N = 5) ± SEM. * represent significant differences with *p*≤0.05, **p≤0.01.

RT^2^-PCR method was used to measure DNA titers in the vaginal tissues collected at 3 and 7 day of infection in mice; the results showed significantly increased titers of HSV-2 in the vaginal tissues of Fas and FasL-deficient mice both at 3 and 7 day of infection (*p*≤0.005) (Fig. 6). Furthermore, we tested for the total numbers of CD4+ and CD8+ T cells and NK cells in the cell suspensions prepared from control and infected mice. In comparison to the uninfected control, all tested strains significantly up-regulated the total numbers of CD4+ T cells and NK cells at day 7 of infection, and the total numbers of CD8+ T cells at day 3 and 7 of infection (*p*≤0.05) (Fig. 7). However, the total numbers of CD4+ T cells in Fas- and FasL- deficient mice at day 7 of infection were significantly lower (*p*≤0.01) in comparison to HSV-2 infected wild type strain at the same time point (Fig. 7A). The total numbers NK cells and CD8+ T cells in the vaginal tissue isolated from Fas- and FasL- deficient mice at day 3 of infection were also significantly lower (*p*≤0.05) (Fig. 7B, C). At day 7 of infection, the total numbers of CD8+ T cells were significantly decreased in both Fas- and FasL-lacking mice (*p*≤0.05) (Fig. 7B) but for NK 1.1+ cells only in Fas-deficient mice (*p* = 0.04) (Fig. 7C).

## Discussion

The goal of this study was to investigate the role of Fas pathway in apoptosis of monocytes during in vitro HSV-2 infection of mouse monocytes and in vivo HSV-2 vaginal infection of mice. Furthermore, we also studied the immunomodulatory role of Fas pathway in monocyte inflammatory response during in vivo HSV-2 infection.

In our study both monocytes and keratinocytes underwent similar levels of apoptosis upon HSV-2 infection at 18 hours post-infection but in contrast to keratinocytes, HSV-2 infected monocytes showed early apoptotic response already at 6 hours post-infection. Release of cytochrome c from mitochondria results in activation of caspase-9 and the effector caspases [16]. Cleavage of caspase 9 was demonstrated to be responsible for apoptosis in CD4+ T cells following exposure to HSV-2-infected fibroblasts [7]. In our study, early induction of apoptosis in HSV-2-infected monocytes was reflected by the significantly higher activation of caspase-9, in comparison to HSV-2 infected keratinocytes. Release of cytochrome c and other apoptotic factors is regulated by Bcl-2 family proteins that form homodimers and heterodimers controlling mitochondrial membrane permeability.

Some Bcl-2 family proteins (Bcl-2 and Bcl-xL) may block apoptosis whereas others (Bax, Bak, and Bid) promote apoptosis by interfering with anti-apoptotic factors [16]. HSV-2 ICP PK increased stabilization of Bcl-2 expression in cultured hippocampal neurons [17], while Bcl-2 down-regulation was associated with induction of apoptosis by HSV-2 in U937 monocytoid cells [8]. Overexpression of Bcl-2 in U937 cells dramatically increased the capability of these cells to sustain a fully productive infection, while protecting against apoptosis induced by HSV-2 [8]. In the previous study we showed that HSV-2 infection up-regulates Bcl-2 expression in keratinocytes and epithelial cells [2]; here we show that HSV-2 infected monocytes did not up-regulate the anti-apoptotic Bcl-2 protein expression, resulting in no compensation for the expression of pro-apoptotic Bax protein. In contrast to our previous study showing that HSV-2 infected mouse epithelial cells and keratinocytes up-regulated anti-apoptotic factors such as Bcl-2, Akt and NF-kB early during infection, thus rendering HSV-2 infected cells resistant to apoptosis induction [2], here we found that HSV-2 infection does not protect mouse monocytes from apoptosis and both infected and uninfected cells are subjected to cell death induction.

It was previously shown [9] that freshly isolated murine peritoneal macrophages underwent apoptosis upon HSV-2 infection, which was associated with the up-regulation of Fas and TNF-receptor 1 (TNF-R1) pathways. However, inhibition of Fas and blocking of TNF-β did not prevent HSV-2-induced apoptosis [9]. In our study HSV-2 monocytes up-regulated FasL during the whole tested period of HSV-2 infection but Fas expression was elevated early during infection on the surface of infected cells, making them sensitive to apoptosis. Later during infection both infected and uninfected monocytes down-regulated Fas expression, although remained sensitive to Fas-induced apoptosis. Furthermore, Fas-deficient monocytes showed decreased apoptosis in a co-culture of HSV-2 infected keratinocytes and monocytes. Therefore, we suggest that during HSV-2 infection, monocytes die early both by mitochondrial and Fas-dependent pathway, while keratinocytes die through mitochondrial pathway of apoptosis later during infection.

Iannello et al. (2011) showed that HSV-1, whose genome is collinear with HSV-2, induced the de novo expression of FasL on the surface of human monocytes and macrophages, which caused death of monocytic cells growing in suspension, but not in monolayers (e.g., macrophages) [18]. The authors also showed that FasL expression on monocytes acts as an immune evasion mechanism by causing the death of interacting human CD4+ and CD8+ T cells, and natural killer (NK) cells [18].

To further study the role of Fas in monocyte apoptosis in vivo, we used a murine model of HSV-2 infection. Vaginal lesions developed during HSV-2 infection are characterized by the increased infiltration of different types of innate immune cells, including NK cells, macrophages and neutrophils within epithelium and the subepithelial layer, eventually leading to disruption of the vaginal epithelium integrity [2]. In the murine model, which we used in this study, we also observed an increased infiltration of monocytes and similarly as for the in vitro model, the monocytes up-regulated FasL and down-regulated Fas expression. Despite the fact that HSV-2 infection in Fas or FasL-deficient mice leads to the increased infiltration of neutrophils to the site of infection [2] we observed decreased infiltration of monocytes at 3 day of infection in comparison to wild-type mice; on the other hand, at 7 day post-infection, an increased percentage of monocytes could be detected within the HSV-2 infected sites in Fas- and FasL-deficient mice in contrast to wild-type mice where lesser infiltration of monocytes was detected. These results indicate that the Fas/FasL pathway is at least partially responsible for the apoptosis of monocytes in vivo early during infection. Previous studies showed that apoptosis was not impaired in HSV-2 infected macrophages isolated from Fas-deficient B6-lpr/lpr mice [9], suggesting the involvement of other apoptotic pathways in these cells. Induction of monocyte apoptosis via Fas/FasL-dependent pathway may be pivotal to attract inflammatory cells within the infection sites, by leading to the production of inflammatory cytokines and chemokines by monocytes.

Monocytes and macrophages represent important cellular elements of the immune system. In response to a viral infection, they release a variety of pro-inflammatory cytokines and chemokines, and recruit inflammatory cells to the site of infection. Activated macrophages phagocytose pathogens and present viral antigens to other immune cells. During HSV-2 infection in vivo, IL-1β facilitates the recruitment of neutrophiles to inflammatory sites [19], [20]. TNF-α stimulates differentiation of NK cells but together with IL-1β also enhances Langerhans cells migration from the epithelium of mice [21]. CXCL1 expression is up-regulated in the vagina and CNS of mice during acute HSV-2 infection [22] and this chemokine specifically targets neutrophils through the receptor CXCR2 promoting chemotaxis and activation of neutrophils [23]. CXC type chemokines, including CXCL9 and CXCL10, are potent chemoattractants for activated T cells, NK cells, monocytes, dendritic cells and B cells [24], [25]. It has been shown that lack of CXCL9 and CXCL10 significantly alters the ability of the host to control genital HSV-2 infection through the mobilization of effector cells to sites of infection [22].

Various studies have suggested an important role of NK cells, as part of an innate immune response, in controlling viral replication in infections such as HSV-2. Ashkar and Rosenthal (2003) showed that *IL-15 (−/−)* mice, which lack NK and NKT cells, were more susceptible to infection [26]. Also NK cell depletion increased the HSV-2 titers in the spinal cord, brain stem and vaginal tissue [27]. Induction of HSV-2 specific CD8+ cytotoxic T cell responses directed towards HSV-2 antigens was shown to be necessary for virus clearance [28], [29]. However, murine models of HSV-2 infected *Cd4−/−* or *Ifng −/−* mice showed that CD8+ cells failed to migrate to the vaginal epithelium in CD4+ deficient or depleted mice [29], which is further reflected in the delayed virus clearance from the vaginal tissue [30]. Taken together, these investigations suggest that an effective immune response against HSV-2 requires the presence of both CD4+ and CD8+T cells, with CD4+ T cells helping to mount the CD8+ T cell anti-viral response. In our study inefficient mobilization of NK cells, CD4+ and CD8+ cells at the site of HSV-2 infection is accompanied by the increased virus titers observed in Fas and FasL deficient mice at day 3 and 7 of infection, indicating a possible delayed virus clearance from the vaginal tissue.

Due to cell-death induction in monocytes during HSV-2 infection and its inflammatory character, the Fas/FasL pathway also appears to be important for further resolution of HSV-2 infection. The source of FasL may consist of HSV-2 infected keratinocytes, up-regulating FasL [2] but also HSV-2 infected monocytes with FasL expression. When stimulated through the Fas pathway, HSV-2 infected macrophages do not only undergo apoptosis but may also up-regulate the production of TNF-α, CXCL9 and CXCL10 - chemo-attractants for activated T cells and NK cells necessary to eradicate local infection and inflammation.

We conclude that apoptotic response of monocytes induced through Fas/FasL pathway during HSV-2 infection plays an important role in mounting local immune response ensuring recruitment of inflammatory and immune competent cells to the site of HSV-2 infection.

These results are important for the development of an effective vaginal microbicides against HSV-2 infection based on the posibility of influencing the local immune responses to HSV-2 infection by modulation of Fas/FasL expression and Fas mediated apoptosis. In addition, in consideration of the results presented in our paper, the Fas receptor may become the direct target of potential therapy aimed at altering the inflammatory milieu in the vaginal compartment.
