# Expression of concern: HSV-2 regulates monocyte inflammatory response via the Fas/FasL pathway

**DOI:** 10.1371/journal.pone.0344636

**Published:** 2026-03-11

**Authors:** The PLOS One Editors

Several concerns have been raised following publication of this article [1]. Specifically:

- The isotype control FACS dot plots shown in Fig 2A (monocytes and keratinocytes panels) and in Fig 3A (Fas+ cells panel) appear to be similar to one another,
- The error bar of the ‘keratinocytes, 48h’ sample in Fig 1A appears to be incomplete.
- In the ‘parent population’ panel of Fig 4B, the upper middle section appears to be missing.
- The animal methodology does not report the number of animals used, the humane endpoints, or the method of euthanasia.

The first author stated that the isotype control FACS dot plots listed above are not the same, that the isotype control Fas+ FACS dot plot shown in Fig 3A is correct, and that the two isotype control dot plots shown in Fig 2A were mistakenly included during figure preparation. An updated Fig 2, with updated isotype control panels in Fig 2A, is provided here as S1 File. The first author provided the FACS analysis reports for both isotype controls shown in Fig 2A (provided here as S2 File) but stated that the original underlying fcs files are no longer available. In the absence of the original fcs files underlying Figs 2A and 3A and given that the isotype controls are required to show the non-specific binding of antibodies, the *PLOS One* Editors consider this concern to be unresolved.

The first author provided full, corrected versions of Fig 1 and Fig 4, provided here in S3–S4 Files.

In regard to the animal experiments, the first author stated that:

- Approximately 140 animals were used during this study.
- Animals were checked and weighed daily by a technician and a researcher, and were observed for neurological symptoms, ulceration of the anogenital area, and inflammation around the vulva.
- Humane endpoints were applied throughout the study; specifically, animals were euthanized when they had lost 20% of their body weight, or had developed gross inflammation of the anogenital area, ulceration, and neurological signs (paralysis).
- Animals were euthanized by isoflurane anesthesia (5% in oxygen), followed by cervical dislocation.
- The original underlying data to support the results in [1] are no longer available.

The *PLOS One* Editors issue this Expression of Concern to notify readers of the above concerns with Figs 2A and 3A which remain unresolved in the absence of the original underlying data.

There is a spelling error in the Materials and Methods section. The ‘Immunofenotyping of Animal Tissues’ subheading should instead be ‘Immunophenotyping of Animal Tissues’.

## Supporting information

S1 File Updated Fig 2, showing corrected monocyte and keratinocyte isotype control dot plots in Fig 2A. (TIF)

S2 File FACS analysis reports underlying both the monocyte and keratinocyte isotype control dot plots shown in Fig 2A in S1 File. (PDF)

S3 File Updated Fig 1, showing the corrected ‘keratinocytes, 48h’ error bar in Fig 1A. (TIF)

S4 File Updated Fig 4, showing the corrected ‘parent population’ panel in Fig 4B. (TIF)

## References

[1] Krzyzowska M, Baska P, Orlowski P, Zdanowski R, Winnicka A, Eriksson K, et al. HSV-2 regulates monocyte inflammatory response via the Fas/FasL pathway. PLoS One. 2013;8(7):e70308. doi: 10.1371/journal.pone.0070308 23922974 PMC3726399

