# Supplementary figures and images for: Expression of concern: HSV-2 regulates monocyte inflammatory response via the Fas/FasL pathway
Source: PLoS One. 2026 Mar 11;21(3):e0344636. doi: 10.1371/journal.pone.0344636 (PMC12978431; doi:10.1371/journal.pone.0344636)

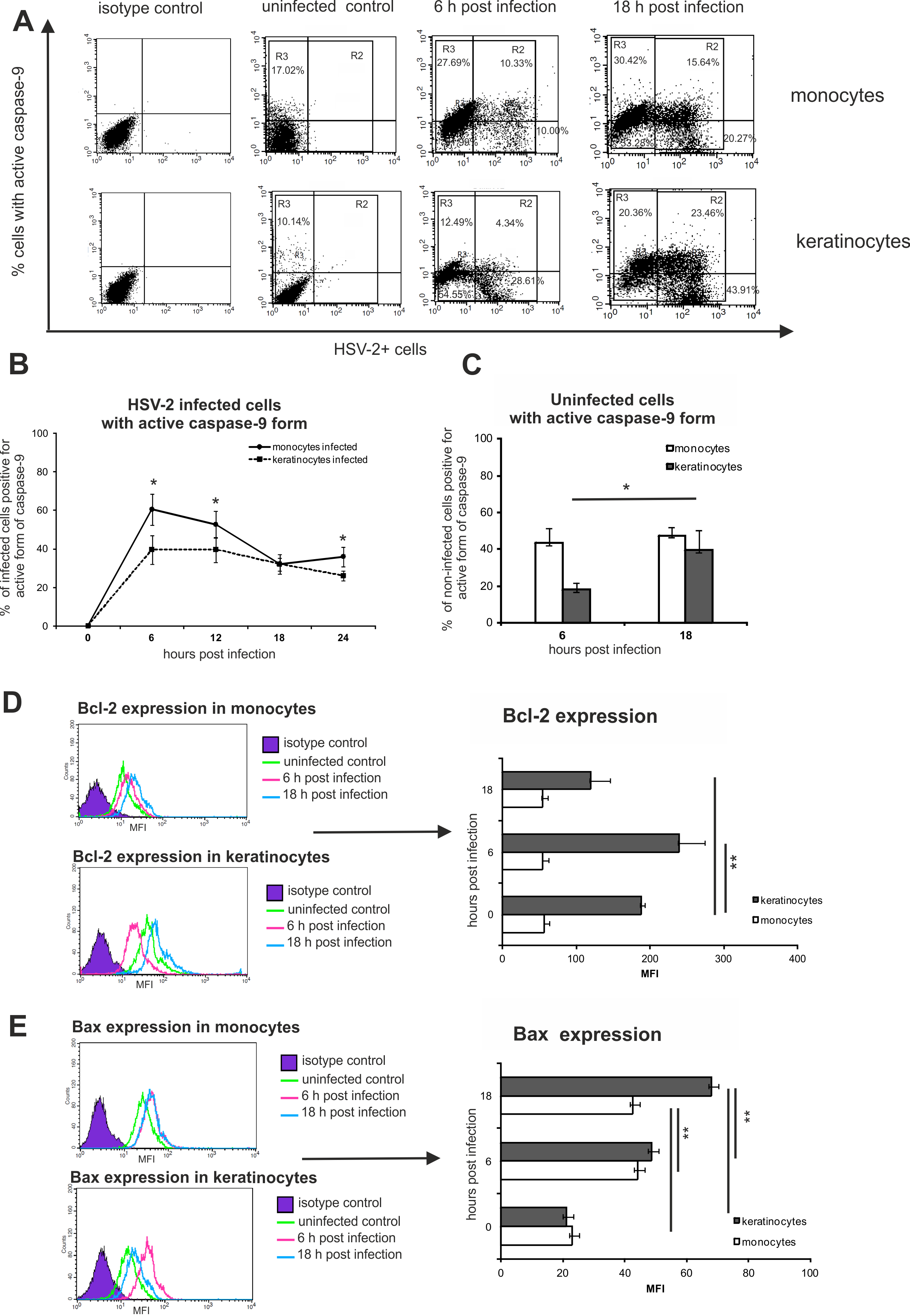

Supplement: S1 File — (TIF) [file pone.0344636.s001.tif]

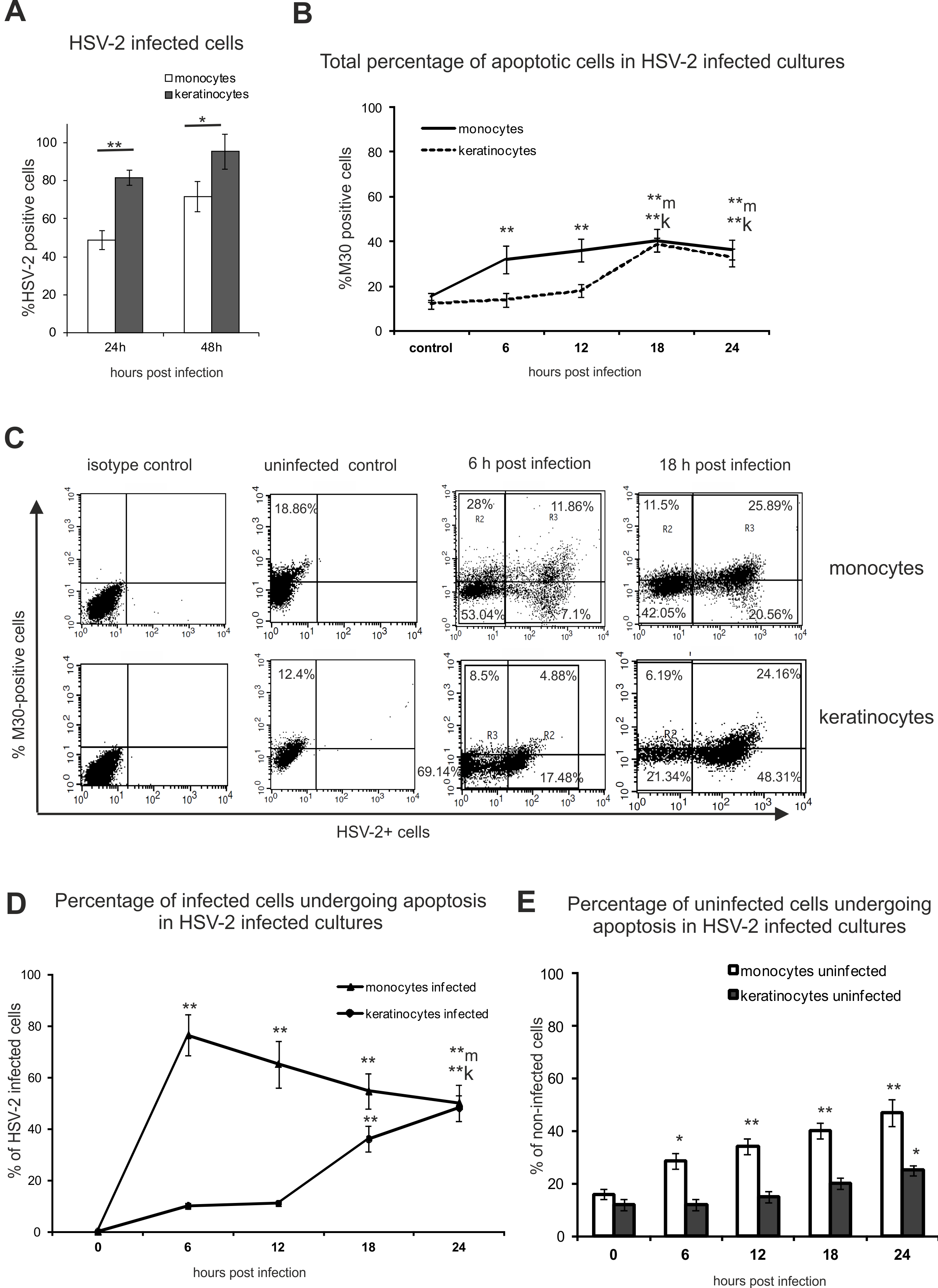

Supplement: S3 File — (TIF) [file pone.0344636.s003.tif]

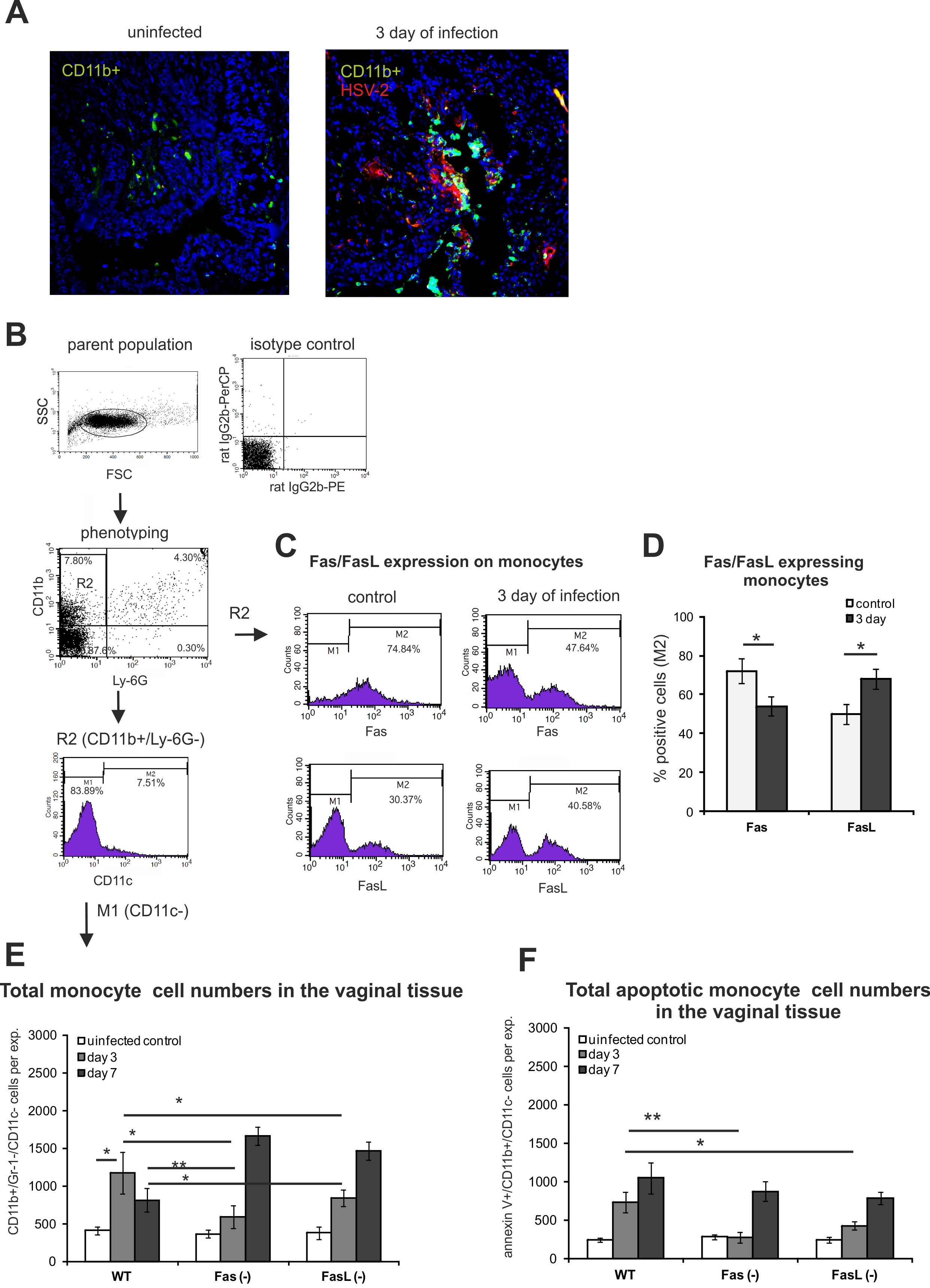

Supplement: S4 File — (TIF) [file pone.0344636.s004.tif]
