# Supplementary material for: Expression of concern: HSV-2 regulates monocyte inflammatory response via the Fas/FasL pathway
Source: PLoS One. 2026 Mar 11;21(3):e0344636. doi: 10.1371/journal.pone.0344636 (PMC12978431; doi:10.1371/journal.pone.0344636)

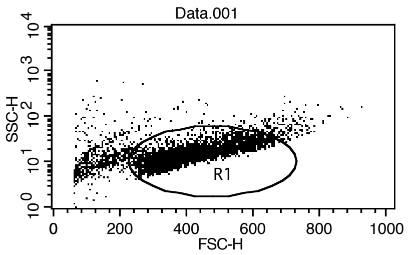

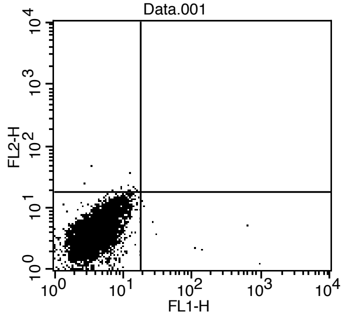

Quadrant Statistics

File: Data.001 Sample ID: mono iso 1

Quad	% Gated	% Total	X Mean	Y Mean
UL	0.14	0.13	10.32	23.62
UR	0.00	0.00	***	***
LL	99.77	90.71	4.15	4.45
LR	0.09	0.08	241.43	5.39

File: Data.002
Sample ID: kera iso 1

Quad	% Gated	% Total	X Mean	Y Mean
UL	0.07	0.06	7.79	21.05
UR	0.00	0.00	***	***
LL	99.89	92.06	3.62	3.22
LR	0.04	0.04	39.91	2.88

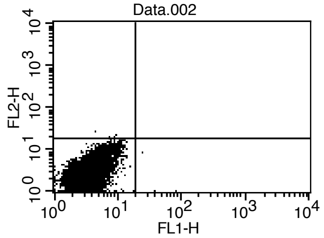

File: M30.003
Sample ID: hsv

Quad	% Gated	% Total	X Mean	Y Mean
UL	0.34	0.28	11.35	27.55
UR	0.32	0.27	292.27	27.98
LL	75.54	62.40	7.07	8.27
LR	23.79	19.65	272.83	5.42

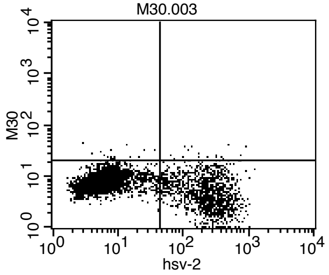

Supplement: S2 File — (PDF) [file pone.0344636.s002.pdf]
